# Draft Genome Sequence of *Pseudonocardia* sp. Strain N23, a 1,4-Dioxane-Degrading Bacterium

**DOI:** 10.1128/genomeA.01240-17

**Published:** 2017-11-02

**Authors:** Norifumi Yamamoto, Daisuke Inoue, Masashi Kuroda, Michihiko Ike

**Affiliations:** a Technology Center, Taisei Corporation, Kanagawa, Japan; b Division of Sustainable Energy and Environmental Engineering, Graduate School of Engineering, Osaka University, Osaka, Japan

## Abstract

*Pseudonocardia* sp. strain N23 is a 1,4-dioxane-degrading bacterium that is capable of utilizing 1,4-dioxane as the sole carbon and energy source. Here, we report the draft genome sequence of strain N23, with a size of 6.5 Mbp, to identify the genes associated with 1,4-dioxane degradation.

## GENOME ANNOUNCEMENT

The possible human carcinogen 1,4-dioxane is an emerging environmental contaminant. Although it has been recognized that 1,4-dioxane is recalcitrant to biodegradation, recent studies have isolated bacterial strains capable of utilizing it as the sole carbon and energy source (1). These findings have suggested that the biological removal of 1,4-dioxane from contaminated environments and wastewater might be possible. However, molecular and enzymatic studies on the mechanism of 1,4-dioxane biodegradation have rarely been performed (2, 3), and even the genome information available is still limited to *Pseudonocardia dioxanivorans* CB1190 and *Mycobacterium dioxanotrophicus* PH-06.

Here, we report the draft genome sequence of *Pseudonocardia* sp. strain N23, a novel strain capable of degrading 1,4-dioxane (N. Yamamoto, Y. Saito, D. Inoue, K. Sei, and M. Ike, unpublished data). Strain N23 has a very high 1,4-dioxane degradation ability and can constitutively express 1,4-dioxane degradation enzymes, unlike the two aforementioned strains.

The genomic DNA of strain N23 was fragmented and prepared into a sequence library using the TruSeq Nano DNA LT library preparation kit (Illumina, San Diego, CA, USA). This library was sequenced by 101-bp paired-end sequencing using the Illumina HiSeq 2500 system, which produced 22,520,910 reads with a yield of 2,275 Mb. From the sequenced reads, the adapter sequences and low-quality value regions were trimmed using the Cutadapt program version 1.1 (https://cutadapt.readthedocs.io/en/stable/) (4) and the Trimmomatic program version 0.32 (http://www.usadellab.org/cms/?page=trimmomatic), respectively. The trimmed reads were assembled onto the draft genome sequence using the Velvet program version 1.2.08 (http://www.ebi.ac.uk/~zerbino/velvet/). The draft genome sequence of strain N23 contains 173 contigs, accounting for a total of 6,542,330 bp (72.5% G+C content), with an *N*_50_ of 183,452 bp and a maximum contig size of 624,394 bp. A total of 6,230 coding sequences (CDSs), 46 tRNA genes, and 3 rRNA genes were predicted within the genome by the Rapid Annotation using Subsystems Technology pipeline.

Tetrahydrofuran monooxygenase genes, which were closely related to those of 1,4-dioxane-degrading *Pseudonocardia* spp., were found in the strain N23 genome. This indicates that these genes are associated with the initial oxidation of 1,4-dioxane. Glycolate dehydrogenase genes involved in the oxidation of glycolate to glyoxylate, an important intermediate in the proposed 1,4-dioxane degradation pathway (2), were also found. These genes may be present possibly for 1,4-dioxane metabolism. The absence of genes associated with toxin production and pathogenicity in the genome of strain N23 may suggest that it is safe for use in environmental applications.

### Accession number(s).

The draft genome sequence and annotation have been deposited in the DDBJ/EMBL/GenBank database under the accession no. BEGX01000001 to BEGX01000173.
